# Unpleasant Food Odors Modulate the Processing of Facial Expressions: An Event-Related Potential Study

**DOI:** 10.3389/fnins.2020.00686

**Published:** 2020-06-30

**Authors:** Danyang Li, Jiafeng Jia, Xiaochun Wang

**Affiliations:** School of Psychology, Shanghai University of Sport, Shanghai, China

**Keywords:** food odor environment, unpleasant odors, recognition of facial expressions, mood coherence effect, vertex positive potential, ERPs

## Abstract

In real-life situations, emotional information is often expressed through multiple sensory channels, with cross-talk between channels. Previous research has established that odor environments regulate the recognition of facial expressions. Therefore, this study combined event-related potentials (ERPs) with a facial emotion recognition task to investigate the effect of food odor context on the recognition of facial expressions and its time course. Fifty-four participants were asked to identify happy, fearful, and neutral faces in an odor context (pleasant, unpleasant or neutral). Electroencephalography (EEG) was performed to extract event-related potentials (ERPs). Behaviorally, unpleasant food odors triggered faster recognition of facial expressions, especially fearful ones. ERP results found that in the early stage, unpleasant food odors within 80–110 ms evoked a larger P100 amplitude than pleasant food odors and no odors, which showed that the unpleasant odor environment promoted the rapid processing of facial expressions. Next, the interaction between odor environment and facial expressions occurred during the middle stage, and the fearful expression evoked a smaller VPP (vertex positive potential) amplitude than the happy and neutral expressions when exposed to the unpleasant food odor environment. This result indicates that unpleasant odor environment consumed fewer cognitive resources when judging fearful expression, showing the promoting effect of mood coherence effect. These findings provided evidence for how people chose odor environments to facilitate the recognition of facial expressions, and highlighted the advantages of unpleasant food odors in communicating emotional information across the olfactory and visual pathways.

## Introduction

For individuals with social attributes, emotional stimulation is one of the most important external signals. Emotion is a general and pervasive affective state (Wood et al., 1990), which is usually characterized by positive and negative valence states and has a profound impact on people’s cognitive processing and behavior. Emotions can be expressed through a variety of media, such as faces, bodies, and voices. As a way to convey important information about an individual’s internal state and intention, the rapid decoding of facial expression is crucial for people in society (Palermo and Rhodes, 2007; Rellecke et al., 2012).

In real-life situations, faces rarely are perceived as single entities and most likely appear within a situational context, which may have a strong impaction how they are perceived (Wieser and Brosch, 2012). Previous research has shown that perceptual processing reflected a dynamic interplay between the environment and the observer, see Pourtois et al. (2013) for a comprehensive review. Therefore, the influence of external situational information causes us to process emotional information rarely only in a single channel, such as visual channel. Emotional information is often expressed through multiple sensory channels, with cross-talk between channels. The cross-channel integration of hearing and vision has been extensively studied. Previous studies involved the reading of a neutral sentence in an emotional tone. Subjects were asked to classify facial expressions. The results showed that emotional voice influenced the emotional recognition of facial expression (de Gelder and Vroomen, 2000). Van den Stock et al. (2009) found that the music played by musical instrumentation can also affect individuals’ emotional recognition of body expressions.

Thus previous research has established that emotional information affects visual processing through auditory channels. Olfactory channels can also transmit emotions, and their emotional information interacts with visual emotional information. Studies have shown that odors can be directly projected to emotion-related brain structures (Zald and Pardo, 1997; Soudry et al., 2011), which is an extremely effective emotional trigger (Adolph and Pause, 2012). Pleasant odor induces positive emotions, while unpleasant odor induces negative emotions (Collet et al., 1997; Robin et al., 1999; Gottfried et al., 2002). Furthermore, odors can effectively influence people’s perception of facial expressions. First of all, there is a lot of physiological evidence that many regions contribute to olfactory processing and may be involved in multimodal emotional integration with vision (Carmichael et al., 1994; Rolls, 2004; based on human and non-human primate data). The amygdala may be involved in multi-sensory emotional integration (Maurage and Campanella, 2013). The orbitofrontal cortex (OFC; Carmichael et al., 1994; based on the macaque monkey) also participates in standard odor-quality coding, which may involve the olfactory-visual synthesis of information (Gottfried and Dolan, 2003; Osterbauer et al., 2005; Park et al., 2010). Behavioral evidence also supports this phenomenon. Olfactory stimuli can have an impact on people’s social preferences. In other words, when study participants are not aware of the odor they are smelling, odor stimuli will significantly affect preferences for pictures of faces (Li et al., 2007). Zhou and Chen (2009) found that subjects tended to interpret ambiguous expressions as more fearful when they smelled sweat that had been collected when people were afraid, but this interpretation was not effective when the facial expression was neutral.

The studies described above focused on biological odors emitted by the body, which convey underlying information and are associated with the emotional state of the transmitter. Much less research has been done on environmental odors (e.g., related to food or to non-specific surroundings), which are the most common in life and affect the perceiver’s visual perception of emotion. A few studies have shown that the arbitrary odor environment around people can affect the perception of facial expressions (Leppanen and Hietanen, 2003; Seubert et al., 2010a, b). Seubert et al. (2010b) found that in the presence of odor stimuli, regardless of the valence of the odor (vanillin or hydrogen sulfide), the recognition of a happy face was slower and less accurate, while the recognition of a disgusted face was faster and more accurate. Another study found that any odor may accelerate the speed of the behavioral response during a facial emotional recognition task (Seubert et al., 2010a). Leppanen and Hietanen (2003) found that happy faces were identified more quickly and accurately in pleasant environments (lemon, strawberry or vanilla) than in unpleasant environments (pyridine) or odorless environments. A recent study also found evidence in favor of a faster or a more accurate recognition of facial expressions of emotions conditional on the different pleasantness (isovaleric acid vs. lilac) of the odor (Syrjanen et al., 2017). The current research on the integration of facial expressions and olfactory emotional information reveals two phenomena. One is that olfactory emotional information will affect the evaluation and judgment of neutral or ambiguous facial expressions. Another is that odor will modulate the processing of facial expressions, but it is unclear whether odor-based modulations occur only for emotionally congruent olfactory-visual stimuli or whether odor has an overall effect on the processing of facial expression. The precise cognitive processes that underlie these effects are unknown and require further investigation.

The multi-sensory interaction of olfactory and visual channels is reflected in behavior, but the time-course and underlying mechanism for independent and dependent processing of the olfactory and visual emotional information remain poorly understood. Additional studies will be necessary to clarify the time-course and physiological mechanism of the interaction between olfactory and visual processing. We therefore used the three-stage theory of emotional processing (Luo et al., 2010; Zhang et al., 2014) as the framework for this study. In the early stage of emotional processing, the reactivity of P100 components (Rossion, 2014) in recognizing facial expressions is easily affected by contextual odors (Adolph et al., 2013). Notably, reports have not been consistent (Leleu et al., 2015b). Odors may thus have an effect on facial processing in the early stages of emotional processing. During the middle stage of emotional processing, the N170 component (Rossion, 2014) and its vertex positive potential (VPP) is particularly sensitive to facial expression (Batty and Taylor, 2003). The N170 amplitude for participants’ recognition of expressions of disgust was smaller in the presence of an unpleasant odor, compared with a pleasant odor (Syrjanen et al., 2018). Studies have also found that VPP components are altered by the odor background (Leleu et al., 2015b). This study found that a pleasant odor increased the VPP amplitude regardless of facial expression, and subsequent exploratory analysis also found that an increased VPP in the unpleasant odor environment at right temporal locations (Leleu et al., 2015b). Some studies have proposed that, during the later stage of emotion processing, the late positive potential (LPP; Olofsson et al., 2008) is sensitive to facial expressions of disgust (Trautmann-Lengsfeld et al., 2013; Leleu et al., 2015b). One study demonstrated that the response of LPP to faces was reduced (Adolph et al., 2013). Another study using threat-related odor found no significant LPP effect (Kastner et al., 2016). Rubin et al. found that anxiety-related sweat increased the effect of late LPP on the processing of neutral and ambiguous, but not angry, faces (Rubin et al., 2012). These studies demonstrated that the integration of olfactory and visual emotional information may not be the simple summation of independent processes, but rather, a complicated synthesis of the information provided by numerous sensory channels. However, current evidence on the concrete time process and mechanism of independent or dependent processing of olfactory and visual emotional information provides conflicting results. Accurate and consistent conclusions have been elusive.

To sum up, existing studies have obtained behavioral and physiological evidence for the interaction of emotional information with that obtained through olfactory and visual channels. However, the conclusions drawn vary widely. The odor materials available focused on biological odors, and the negative face materials were mostly disgust expressions. However, some studies have shown that the context created by arbitrary surrounding odorants can affect the perception of facial expressions (Leppanen and Hietanen, 2003; Seubert et al., 2010a, b). Moreover, as a kind of non-bodily odor, food odor can often induce different emotional states in life, and its influence on the processing of facial expressions has not been studied. In addition, fear expression is an important non-verbal form that contains information about potential threats and tends to attract people’s attention, helping to reduce the possibility of being hurt (LeDoux, 1996; Ohman, 2005). Based on this fact, this study started from environmental odors and creatively selected food odors as the odor material and fear expressions as the negative face material. The odor was also combined with the face to establish combinations of positive (pleasant odor)-positive (happy face), negative (unpleasant odor)-negative (fearful face), neutral (no odor)-neutral (neutral face). The aim was to explore the physiological mechanism underlying independent and dependent modes of processing olfactory and visual emotional information, and to examine the effect of emotional coherence in olfactory and visual domains. In addition, we wanted to explore whether food odors, which are common in our lives and can convey different emotional valences, can help us to regulate and recognize visual stimuli. Based on the results reported to date, we hypothesized that facial expression and odor will be processed independently during the early stage of emotion recognition. During the middle stage of emotion recognition, the N170 and VPP components, which are sensitive to facial expression, allow for dependent processing of odor and facial expression.

## Materials and Methods

### Participants

Fifty-four participants (26 men, 28 women; mean age ± SD: 22.72 ± 2.15; range: 18–30 years) took part in the experiment. No subject reported any psychiatric or neurological disorder, nor did any subject report history of an acute nasal infection or allergy that might affect the sense of smell. All subjects had a normal sense of smell. All subjects were right-handed and had normal or corrected-to-normal visual acuity. All subjects were provided written informed consent and were compensated for their participation. The study was conducted according to the Declaration of Helsinki and approved by the ethics committee of the Shanghai University of Sport (102772019RT004).

### Materials

#### Facial Materials

**FIGURE 1:**
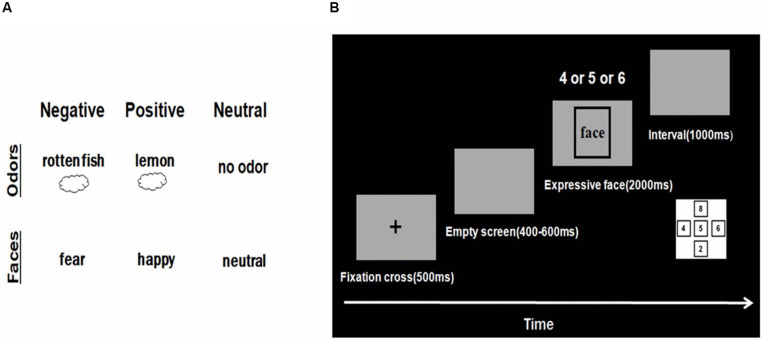
Materials and Procedure. **(A)** Representative odors and faces used in the experiments. **(B)** Time sequence for the experimental trials. Subjects first saw a fixation cross for 500 ms. Next, an empty screen appeared for 400–600 ms. Then a picture was presented for 2000 ms, followed by presentation of an empty screen for 1000 ms before the subsequent trial was initiated. The study had a 3 × 3 factorial design, resulting in the presentation of 9 odor–face combinations (each consisting of 20 trials).

Pictures from the native Chinese Facial Affective Picture System (CFAPS) were compiled by Gong et al. (2011). We chose 120 facial pictures (60 Women and 60 men) of three facial expressions (fear, neutral and happy). Twelve male students and eight female students (average age, 23.32 years) rated the degree of pleasantness, intensity, and arousal of each expression on a 9-point self-assessment manikin (SAM) scale. Finally, 60 pictures of 3 emotions (happy, fearful, and neutral) were screened (20 pictures for each emotion). Face gender was balanced between groups. Emotional intensity was >5.5, with no significant difference between groups [*F*(2,38) = 2.642, *p* = 0.228, η*p*^2^ = 0.051]. Degree of arousal did not differ significantly among groups [*F*(2,38) = 2.642, *p* = 0.08, η*p*^2^ = 0.085]. Valence differed significantly among groups [*F*(2,38) = 171.744, *p* < 0.001, η*p*^2^ = 0.858] (Table 1). Valence was greater for happy expressions, compared with neutral expressions (*p* < 0.001) and fear expressions (*p* < 0.001). Valence was greater for neutral expressions than for expressions of fear (*p* < 0.001). The mean identification rate reached >80% (Ebner et al., 2010). All images were gray scale, with consistent brightness and 385 × 513 pixels. Images were 9 × 12 cm in size, with a dark gray background. Participant’s eyes were 75 cm from the computer monitor. The hair and ears in each image had been removed to prevent any confounding effect on emotion recognition (Figure 1A). For the formal experiments, images were mixed and presented randomly.

**TABLE 1:** The SAM ratings of emotional pictures.

**Emotion type**	**Valence, M ± SD**	**Arousal, M ± SD**	**Intensity, M ± SD**
Happy	7.01 ± 0.87	5.20 ± 0.51	5.85 ± 0.24
Fear	5.02 ± 0.68	5.18 ± 0.31	5.75 ± 0.22
Neutral	2.88 ± 0.52	4.94 ± 0.34	5.74 ± 0.22

#### Odor Materials

We selected lemon odor, rotten fish odor and air odor as odor stimuli. Essential oil smelling of lemon (96+% mixture of *cis* and *trans*, purchased from Sigma-Aldrich) was used as the pleasant food odor. Essential oil smelling of rotten fish (fish flavor oil, Givaudan Inc., Geneva, Switzerland) was used as the unpleasant food odor (Boesveldt et al., 2010). Essential oils were diluted with mineral oil (50%, v/v) and 1,2-propanediol (39%, v/v), respectively. The neutral group was tested in an odor-free environment (Figure 1A). In order to select pleasant and unpleasant odors, we asked 11 raters to smell 9 specific food odors (vanilla, apple, lemon, durian, chocolate, vinegar, alcohol, rotten fish, and garlic) and then rate their pleasantness, intensity, and arousal on a 9-point self-assessment manikin (SAM) scale. Then the odors with the highest and lowest pleasantness scores were selected as pleasant and the unpleasant odor. Many previous studies have used lemon and rotten fish odors as pleasant and unpleasant odor, respectively (Chen and Dalton, 2005; Damjanovic et al., 2018). Lemon odor (*M* = 5.90, *SD* = 0.70) and rotten fishy odor (*M* = 2.27, *SD* = 0.90) differ significantly in terms of pleasantness (*p* < 0.001), but not in degree of arousal (*p* = 0.648). The degree of dilution was selected to achieve moderate odor intensity sufficient to ensure that the odor concentration remained relatively constant.

We used a TLDQ-806 basic air odor diffuser to emit ambient odor. The device functions similarly, to an atomizer. The odor is provided in the form of an aerosol. The odor release mechanism was set to emit the odor at 20 min intervals. Based on previous studies on the emotional and memory effects of odors, environmental odor diffusion has proven to be an effective method of stimulus delivery (Ludvigson and Rottman, 1989; Gilbert et al., 1997; Herz, 1997).

### Experimental Apparatus

A Dell desktop computer and a HP laptop were used in the experiment. The Dell desktop computer was used to present the pictures of faces used for the study. The CPU frequency of the laptop was 1.6 GHz, and the operating system was Windows 10. Images were displayed on the Dell desktop computer screen with a refresh rate of 100 Hz and resolution of 1024 × 768 pixels. A laptop was used to record event-related potentials (ERPs). ERPs were recorded using a device manufactured by Brain Products (Germany). Electroencephalography (EEG) signals were collected using 64 conductive electrode caps positioned in accordance with the international 10–20 standard system. Silver/silver chloride electrodes were used. Horizontal and vertical EEGs were recorded. The horizontal EEG electrode was attached lateral to the right eye; the vertical EEG electrode was attached inferior to the left eye. EEG signals were digitalized at a sampling rate of 1000 Hz (band-pass filter: 0.05–100 Hz). Electrode impedance was maintained below 10 kΩ. EEG data were analyzed with Brain-Vision Analyzer software.

### Study Design

The experimental protocol had a factorial 3 × 3 mixed model design, with odor environment (pleasant, unpleasant, no odor) as the between-subjects variable. The within-subject variable was facial expression (happy, fearful, and neutral). There are 19 people in the pleasant odor group (10 females, 9 males), 17 people in the unpleasant odor group (9 females, 8 males) and 18 people in the no odor group (9 females, 9 males).

### Procedure

All experiments were performed in the same laboratory, which had been properly ventilated prior to odor diffusing. And there was a time interval of at least 4 h between the end of one participant’s test and the beginning of the next participant’s test. During this time, the room was ventilated to ensure that the remaining odor from the previous test will not affect the subsequent experiment. Participants did not see the odor diffuser during the formal experiment.

Participants were divided into three groups and randomly matched into different odor environments. At the beginning of the experiment, each participant was asked if they were aware of the odor in current environment through an instruction. And if they said they smell it, they would use a 7-point scale to rate the pleasantness, intensity, and arousal of the odor on a scale from 1 to 7, ranging from extremely unpleasant to extremely pleasant, from extremely weak to extremely strong, or from not at all arousing to extremely arousing.

Participants sat comfortably in an armchair in front of a computer screen before pressing a button to begin the task. Each participant wore an electrode cap during ERP recording. Head movement was stabilized by a chin rest. The experimental task consisted of three blocks, and each block incorporated 60 trials, resulting in 180 trials in total. In every block, each face was randomly presented once, with a total of 60 faces. In the formal experiment, each face was repeated three times. Participants were shown a series of images of faces, one face at a time. Each facial stimulus indicated a fearful, happy, or neutral emotional state. During each trial, a centrally fixed plus sign was displayed on the screen for 500 ms. This presentation was followed by an empty screen, which remained for 400–600 ms. A face was then displayed for 2000 ms (Figure 1B). Participants were asked to judge whether a face was happy, fearful, or neutral. If the face was happy, participants need press “4” on the keypad with the right index finger. If it is fearful, participants need press “6” on the keypad with the right ring finger. If it is neutral, participants need press “5” on the keypad with the right middle finger. If no response was recorded during the 2000-ms period allotted, the computer automatically presented next trail. An empty screen was presented for 1000 ms between trials. The participants’ hand responses were balanced. Before the formal trials, a small amount of trials were conducted to familiarize the participants with the experimental process. Study participants had the opportunity to take a 1 min break before proceeding to the next block. Each block lasted about 5 min. Total test time was approximately 20 min.

After completing the facial recognition tasks, subjects rated the odor in terms of pleasantness, intensity, and arousal with a 7-point scale. Before and after the facial recognition tasks, the subjects were asked to score the properties of the odor environment in order to compare their subjective feelings on the properties of the odor environment before and after the key pressing task, so as to ensure that there was no difference in the subjective feelings caused by the odor environment during the whole experiment.

### Data Analysis

E-Prime 2.0 software was used to collect behavioral data, such as accuracy and reaction time for performance of a facial emotion recognition task in various odor environments. Data were imported into Excel for preprocessing. Data falling beyond three standard deviations of the mean were excluded. The SPSS 22.0 (IBM, Somers, United States) statistical software package was used to conduct two-way (odor environment × facial expression) repeated-measures analysis of variance (ANOVA). The scores of pleasantness, intensity, and arousal of three odor environments obtained before and after the experimental task were analyzed by the 3 (odor environment: pleasant, unpleasant, and no odor) × 2 (order: before and after) repeated-measures multivariate analysis of variance (MANOVA).

The ERP data collected by Brain Product Recorder software were imported into Brain-Vision Analyzer 2.0 for offline processing and analysis. First, the data obtained with the reference electrode were converted. TP9 and TP10 were used as references to replace the original reference electrode (FCZ). Independent component analysis (ICA) was used to semi-automatically remove ocular artifacts. Next, we removed line noise with a 50 Hz notch filter. The data were filtered with a 30 Hz low-pass cutoff and a 0.5 Hz high-pass cutoff, respectively. Segmentation was performed according to the study protocol. With the target stimulus as the zero point, the time range for analysis was set to range from −200 ms to +800 ms. A time window ranging from 200 to 0 ms before the stimulus was selected as the baseline for baseline correction. Data with amplitude greater than ±80 μV were automatically removed.

Inferential analyses were performed on mean amplitudes for distinct components that have been shown to be associated with the processing of facial expression (e.g., Williams et al., 2006). P100, N170, VPP, and LPP were selected for statistical analysis. Electrode points and time windows were selected according to topographic maps and references (Leleu et al., 2015b; Muller and Gundlach, 2017; Syrjanen et al., 2018). Electrodes PO7, PO8, and OZ were used to analyze P100 (80–110 ms). PO3 and PO4 were used to analyze N170 (130–180 ms). P3, P4, and PZ were used to analyze LPP components (320–480 ms). Electrodes C3 and C4 were used to analyze VPP components (130–180 ms; George et al., 1996). Average ERP waveform amplitude was analyzed with SPSS 22.0 (IBM, Somers, United States) using repeated-measures ANOVA. The two factors analyzed were odor environment and facial expression. *P*-value (two-tailed) < 0.05 was considered statistically significant. The Greenhouse–Geisser method was used to correct *p*-values in the case of statistical significance that did not meet the Spherical test. The Bonferroni method was used to assess multiple comparisons.

## Results

### Baseline Odor Ratings

**FIGURE 2:**
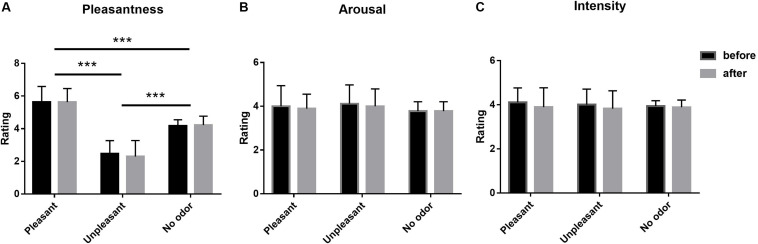
Mean pleasantness **(A)**, arousal **(B)**, and intensity **(C)** ratings of the olfactory environment, as rated on a 7-point scale before and after each experiment (Error bars represent mean SE). ***p < 0.001.

A 3 × 2 (odor environment × order) repeated-measures multivariate analysis of variance (MANOVA) was conducted for ratings of odor pleasantness, intensity, and arousal at the beginning and end of the experiment across three odor environment, and there were three dependent variables (pleasantness, intensity, and arousal). The results of pleasantness showed a significant main effect at odor environment [*F*(2,106) = 155.476, *p* < 0.001, η*p*^2^ = 0.753]. *Post hoc* tests indicated that the pleasantness score of pleasant odor environment was greater than that of no odor environment (*p* < 0.001), and that of no odor environment was greater than that of unpleasant odor environment (*p* < 0.001). No main effect of order [*F*(1,53) = 0.072, *p* = 0.789, η*p*^2^ < 0.01] was found, nor was there a significant interaction between order and odor environment [*F*(2,106) = 0.209, *p* = 0.811, η*p*^2^ < 0.01]. The results of intensity showed that none of the effects were significant. For the main effect of odor environment, *F*(2,106) = 0.216, *p* = 0.806, η*p*^2^ < 0.01; for the main effect of order, *F*(1,53) = 1.398, *p* = 0.240, η*p*^2^ < 0.05, and for the interaction, *F*(2,106) = 0.144, *p* = 0.866, η*p*^2^ < 0.01. The results of arousal revealed no significant main effects of odor environment [*F*(2,106) = 2.373, *p* = 0.098, η*p*^2^ < 0.05] or order [*F*(1,53) = 0.875, *p* = 0.352, η*p*^2^ < 0.01], and no significant odor environment-order interaction [*F*(2,106) = 0.019, *p* = 0.982, η*p*^2^ < 0.01) (Figure 2).

### Behavioral Results

**FIGURE 3:**
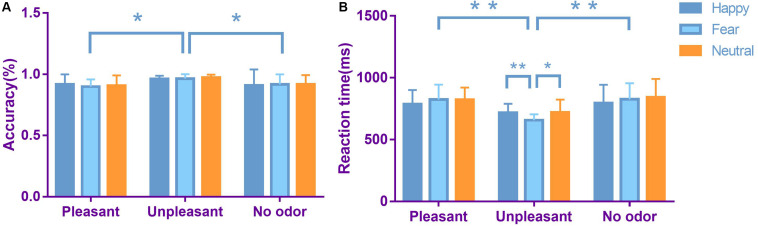
Average accuracy **(A)** and average reaction time **(B)** in each odor × expression condition (bars represent SE). *p < 0.05, **p < 0.01.

We performed a 3 × 3 (odor × expression) repeated-measures ANOVA to evaluate the accuracy of performance on facial emotion recognition tasks (Figure 3). The main effect of odor was significant [*F*(2,106) = 5.480, *p* = 0.007, η*p*^2^ = 0.177]. Accuracy was greater in an unpleasant odor environment, compared with a pleasant odor environment (*p* = 0.011) and a neutral odor environment (*p* = 0.029). There was no significant main effect of expression [*F*(2,106) = 0.123, *p* = 0.884, η*p*^2^ < 0.01]. The interaction between face and odor was not significant [*F*(4,212) = 0.352, *p* = 0.842, η*p*^2^ = 0.014].

We performed a 3 × 3 (odor × expression) repeated-measures ANOVA for reaction time on facial emotion recognition tasks (Figure 3). The main effect of expression was significant [*F*(2,106) = 4.027, *p* = 0.021, η*p*^2^ = 0.073]. Reaction time was shorter for happy faces, compared with neutral faces (*p* = 0.035). There was no significant difference between happy faces and fearful faces. The main effect of odor was significant [*F*(2,106) = 7.381, *p* = 0.002, η*p*^2^ = 0.224]. Reaction time was shorter for unpleasant odors, compared with pleasant odors (*p* = 0.006) and neutral odors (*p* = 0.003). The interaction between face and odor was significant [*F*(4,212) = 4.148, *p* = 0.004, η*p*^2^ = 0.140]. Simple effect analysis showed that, in an unpleasant odor environment, reaction time was faster for identification of fearful faces, compared with happy faces (*p* = 0.005). Reaction time was also faster for fearful faces, compared with neutral faces (*p* = 0.013).

### ERP Results

A 3 × 3 repeated-measure ANOVA was performed to evaluate the mean amplitude of EEG components (P100, N170, VPP, and LPP) induced by odors and facial expressions.

#### P100

**FIGURE 4:**
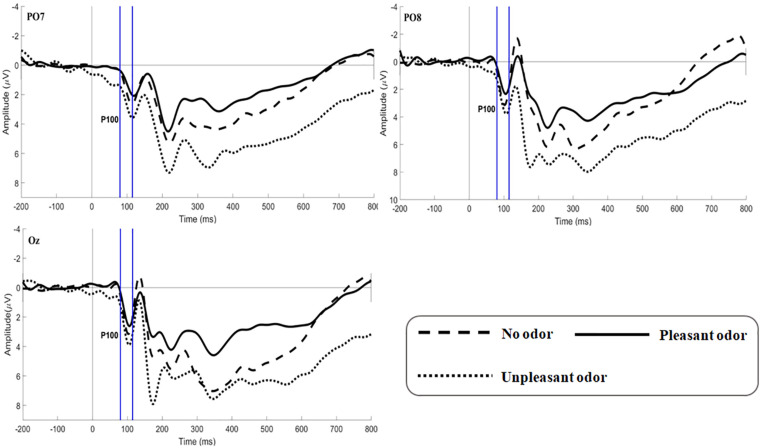
Grand average ERP waveforms of P100 for no odor, pleasant odor and unpleasant odor recorded at electrodes PO7, PO8, and Oz.

The main effect of expression was not significant [*F*(2,106) = 1.361, *p* = 0.260, η*p*^2^ = 0.026]. The main effect of odor was significant [*F*(2,106) = 5.179, *p* = 0.009, η*p*^2^ = 0.169; Figure 4]. Unpleasant odor induced larger P100 amplitude than pleasant odor (*p* = 0.013). Unpleasant odor induced larger P100 amplitude than no odor (*p* = 0.037). The interaction between expression and odor was not significant [*F*(4,212) = 0.544, *p* = 0.704, η*p*^2^ = 0.021].

#### N170

The main effect of odor did not reach statistical significance [*F*(2,106) = 2.149, *p* = 0.127, η*p*^2^ = 0.078]. The main effect of expression was not significant [*F*(2,106) = 0.793, *p* = 0.455, η*p*^2^ = 0.015]. The interaction between expression and odor was not significant [*F*(4,212) = 0.586, *p* = 0.674, η*p*^2^ = 0.022].

#### VPP

**FIGURE 5:**
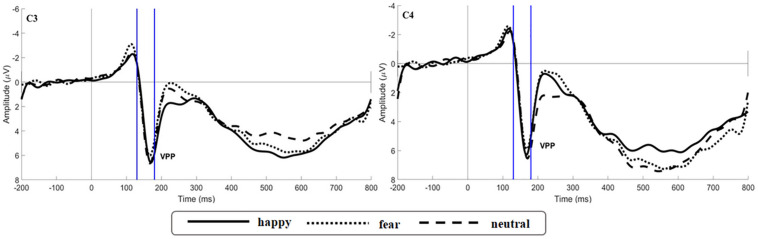
Grand average ERP waveforms of VPP for happy, fear and neutral conditions recorded at electrodes C3 and C4.

**FIGURE 6:**
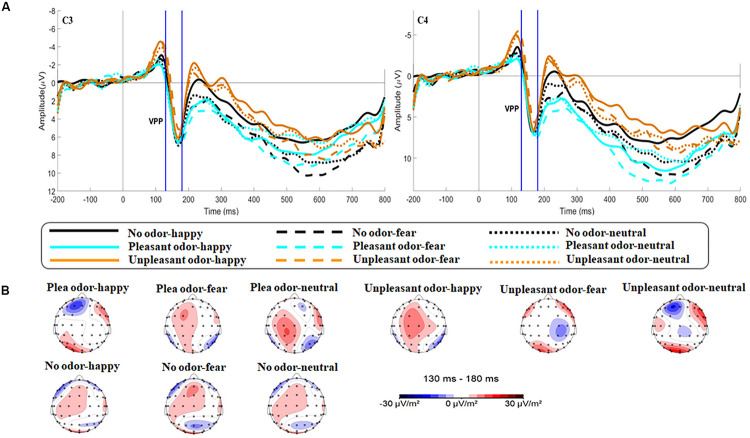
The amplitude and topography for VPP for each odor-face condition. **(A)** Interaction of expression and odor context at electrodes C3 and C4 for VPP. The area between the two blue dotted lines indicates the time window (130–180 ms) of the VPP component. **(B)** Brain topography of VPP for each odor-face condition.

**FIGURE 7:**
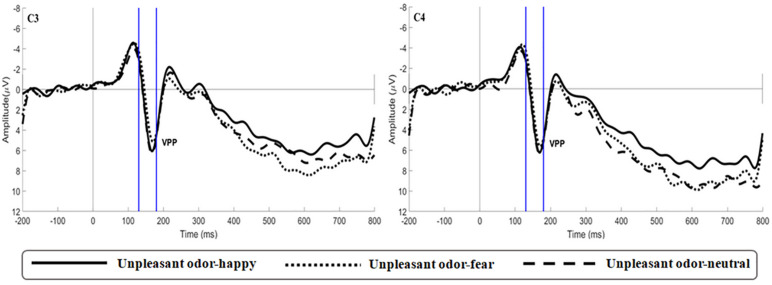
Average ERP waveforms of VPP for happy, fear and neutral in the unpleasant odor contexts recorded at electrodes C3 and C4.

The main effect of expression was significant [*F*(2,106) = 6.659, *p* = 0.002, η*p*^2^ = 0.115; Figure 5]. While happy expressions induced larger VPP amplitude than fearful expressions (*p* = 0.007), neutral expressions induced larger VPP amplitude than fearful expressions (*p* = 0.030). The interaction between expression and odor was significant [*F*(4,212) = 2.922, *p* = 0.025, η*p*^2^ = 0.103; Figure 6A] (the topographic map is shown in Figure 6B). Simple effect analysis showed that the VPP amplitude induced by the evaluation of happy expressions in an unpleasant odor environment was greater than that observed for the evaluation of fearful expressions (*p* = 0.006). The VPP amplitude induced by the evaluation of neutral expressions was greater than that induced by the evaluation of fearful expressions (*p* = 0.009; Figure 7). The main effect of odor was not significant [*F*(2,106) = 0.777, *p* = 0.465, η*p*^2^ = 0.030].

#### LPP

None of the effects were significant for the interaction [*F*(4,212) = 0.781, *p* = 0.527, η*p*^2^ = 0.030], for the main effect of odor [*F*(2,106) = 0.633, *p* = 0.535, η*p*^2^ = 0.024], or for the main effect of facial expression [*F*(2,106) = 0.714, *p* = 0.477,η*p*^2^ = 0.014].

## Discussion

This study aims to explore the influence of olfactory information on visual emotional perception, as well as the associated time course and underlying mechanism. We sought to determine whether olfactory emotional information, which is essential to our life and survival, can promote or inhibit our recognition of visual emotions, and to provide support for research on the multi-sensory crossover pattern. We obtained behavioral and physiological evidence that odor is a clue that can strongly affect human emotions. Odor not only causes the emotions conveyed by transmitters but also regulates people’s visual emotional perception.

### Effect of Odor on the Recognition of Facial Expression

The behavioral results of this study showed that the participants recognized fearful faces more quickly than other expressions when exposed to unpleasant odors. This finding was consistent with the findings of many studies. For example, in an odor environment that was consistent with the emotions expressed by a face (for instance, unpleasant odor paired with distasteful facial expression), people recognized facial emotions faster and more accurately than they did under other odor-face conditions (Leleu et al., 2015a). Since human sense of smell was highly adaptive (Dalton, 2000), we were concerned about whether the adaptability of olfaction could change participants’ perception of odor pleasantness, intensity and arousal during the course of the experiment, thus affecting the final result. When we compared ratings of odor pleasantness, intensity, and arousal obtained before vs. after the experiment, there was no significant difference in the rating of odor pleasantness in any of the three odor environments, no significant difference in the rating of intensity, and no significant difference in the degree of arousal. Therefore, the potential influence of changes in odor pleasantness, intensity, and arousal on odor adaptability was excluded from analysis. Moreover, there was no significant difference in the ratings of intensity and arousal across the three odor environment before and after the experiment, which proved that we controlled the concentration of the three environmental odors properly. In response to other similar results, Leppanen and Hietanen (2003) proposed, that in a pleasant odor environment, by increasing the availability of positive emotions, the recognition of happy faces was improved, thus enhancing the perceptual processing of emotional consistency in facial signals. In this study, an unpleasant odor environment (especially the odor of rotten food) increased the availability of negative emotions and thus promoted the recognition of fearful faces. The results also conformed to the view that the processing of emotionally consistent information was facilitated by cross-modal communication (Seubert et al., 2010a). A discrepancy in habituation rate (Steinberg et al., 2012) may also underlie the observed results. The habituation rate was used to explain this result, which means that fearful faces paired with an incongruent pleasant odor habituate slower across multiple trials.

In addition to this interaction between odor and facial expression, the behavioral results obtained also demonstrated an overall effect of odor on the processing of facial expression. Participants responded more quickly to faces in environments with unpleasant smells, regardless of the emotional content of the face. This finding was consistent with the view that emotional odors increase emotional arousal and thus affect overall performance (Bensafi et al., 2002), with unpleasant odor likely to increase emotional arousal. One reason why the emotional consistency effect was not observed with accuracy may be that the emotional intensity in our selected facial materials was >5.5, with the degree of recognition reaching 80%. It was relatively easy for people to recognize expressions, so the difference in accuracy observed in various odor environments was small.

### The Stages of Facial Expression Processing

Measurements of the VPP showed that happy and neutral faces produced larger VPP amplitudes than did fearful faces, starting 130 ms after the stimulus was administered. This result indicated that VPP component was more sensitive to happy and neutral faces in the middle stage of emotional information processing, reflecting the processing advantage of non-negative expressions. This finding was inconsistent with the results of a previous study, which demonstrated that the VPP component was more sensitive to negative emotional information (Leleu et al., 2015b). This could perhaps be explained by differences between studies in stimulus materials or of the chosen reference electrodes. Recent studies suggested that N170 may be sensitive to the detection of emotional faces, but others suggested that N170 may be unrelated to the processing of facial emotion (Eimer et al., 2008; Kiss and Eimer, 2008). Our results confirmed that the influence of facial emotion was not reflected in N170 measurements. N170 components may be sensitive, not to the detection of facial expression, but, rather, to the structural coding of faces (Bentin et al., 1996).

The ERP results we observed showed that late LPP components did not adjust for facial expression. This was consistent with the results of Syrjanen et al. (2018). Previous work has shown that LPP components reflect the deep processing and classification of stimuli (Kutas et al., 1977; Pritchard, 1981). LPP components also reflected the amount of psychological resources invested by a given individual (Kok, 1997). Our results suggested that the individual was no longer deeply processing facial expression during the later stages of emotion recognition. The facial expressions in the selected experimental materials were easy to recognize, so study participants were able to process them quickly, finishing during the early stage of facial recognition.

### Time-Course for the Effect of Olfactory Environment on the Processing of Facial Expression

The ERP results obtained demonstrated that the influence of odor environment on the processing of facial expression may be divided into two stages. During the first stage, odor has a nonspecific effect on the early stages (P100) of facial expression processing. In other words, regardless of the emotional content of a given facial expression, 80 to 110 ms after the appearance of a face, and unpleasant odor environment enhanced the overall recognition of facial expression, in comparison to an odor environment that was neutral or pleasant. This result was consistent with the findings of Syrjanen et al. (2017), these authors found that the unpleasant odor seemed to speed up participant’s cognition of facial expression, in comparison to an odor environment that was neutral or pleasant (Syrjanen et al., 2017). This effect was consistent with previous research showing that odor had a nonspecific effect on the processing of facial expressions during at least one stage of olfactory-visual integration (Forscher and Li, 2012; Rubin et al., 2012; Leleu et al., 2015b), as well as our own hypothesis. The reason for this result may be that the olfactory system responds more quickly and accurately to ecologically relevant stimuli that signal potential danger (Boesveldt et al., 2010), thus making people more responsive to unpleasant odors.

During the second stage, odor environment had a specific influence on the processing of facial expressions. This corresponded to the VPP during the middle stage of emotional processing (Luo et al., 2010): 130 ms after the appearance of a facial expression, the odor environment specifically regulated the recognition of facial emotion based on whether it was consistent or inconsistent with its emotional valence. VPP amplitude was smaller when evaluating a fearful expression that was consistent with the odor valence in an unpleasant odor environment than when evaluating other combinations of odors and facial expressions. This may be because, in an unpleasant odor environment, a happy expression that was inconsistent with its valence caused the subject to experience greater emotional conflict, thus consuming more emotional attention resources, which extended behavioral reaction time. A topographic map of the brain during the VPP component showed brain activation in the frontal parietal lobe, which was involved in cross-modal integration. Brain activation was significantly lower during the evaluation of fearful expressions in an unpleasant odor environment, compared with other odor-expression combinations (Figure 6B). This was consistent with the research reported by Seubert et al. (2010b). For fearful expressions, consistent odor exposure may promote the processing of facial expressions and result in decreased levels of activation. Syrjanen et al. (2018) showed that the expression of disgust was influenced by the odor environment, with a smaller N170 amplitude in the presence of an unpleasant odor. Numerous studies have shown that N170 and VPP components were sensitive to facial emotions. N170 and VPP were generated by the same dipole in the fusiform region (Joyce and Rossion, 2005). This region was also highly sensitive to cross-modal integration (Park et al., 2010; Gerdes et al., 2014), so it was easier to observe evidence of multi-sensory integration when studying these two components. The rate of habituation may be linked to the enhancement of multisensory information with consistent valence. When fearful faces pair discordantly with a pleasant odor, there was a conflict that leaded to slower adaptation across multiple trials. Some researchers have proposed that the processing of emotional consistency information was characterized by cross-modal promotion (Seubert et al., 2010a). This hypothesis was confirmed by our research results.

In this study, the LPP component did not show any effect of odor. Examining this result in the context of previous research was complicated. Previous studies have shown that emotional odor had no effect on LPP (Leleu et al., 2015b; Syrjanen et al., 2018). Other studies have demonstrated increased LPP amplitude in the context of sweat odor (Rubin et al., 2012). The inconsistency of these results may reflect the use of different experimental materials or electrodes. Therefore, studies with larger sample size or highly accurate processing methods were needed to resolve these discrepancies.

### Limitations and Future Directions

The odor environment was taken as an intergroup factor in our study. Each subject was randomly entered into an odor background, which controlled for the effect of learning across trials. Furthermore, we acknowledged that empirical ERP data did not allow researchers to draw accurate conclusions about the brain regions involved, and further research is needed to solve this problem. In future studies, besides making up for the shortcomings of the this study, functional magnetic resonance imaging technology may be used to explore cortical mechanisms, such as the connections between brain regions activated by specific combinations of stimuli. Not only that, different ages and cultures can influence people to make different behavioral and psychological reactions (Ortlieb et al., 2020). Future studies may also explore age-related differences in olfactory-visual processing. In addition, culture also plays an important role in odor and food perception (Ferdenzi et al., 2011, 2013), so future research can assess current results from a cultural perspective.

## Conclusion

In conclusion, both behavioral and physiological evidence suggested that food odor environment may regulate the recognition of facial expressions. Unpleasant food odor environment promoted the recognition of facial expressions and consumed fewer attention resources when judging fear expression, showing the promoting effect of mood coherence effect. Based on the ERP results obtained, the overall effect of odor environment on facial expression and the processing of facial expression appear to start during the early stage and middle stage of emotion recognition. First of all, exposure to an unpleasant odor within 80–110 ms will enhance the recognition of facial expressions. Next, the VPP component during the middle stage showed that people will give more cognitive resources to pleasant and neutral faces, and the pre-processing of faces would be affected by odor environment, which began 130 ms after presentation of the face picture. At this point in processing, an unpleasant food odor symbolizing danger will enhance the perception of a fearful expression in response to danger. LPP did not show any effect of odor and facial expressions. The dynamic interaction between olfactory emotional information and facial expressions and the time-course of processing provide evidence for multi-sensory integration during processing. The highly adaptive response to unpleasant stimuli is evidence of olfactory-visual multimodal integration, which promotes appropriate behavior in the presence of danger.

## Data Availability Statement

All datasets presented in this study are included in the article/Supplementary Material.

## Ethics Statement

The studies involving human participants were reviewed and approved by the ethics committee of the Shanghai University of Sport. The patients/participants provided their written informed consent to participate in this study.

## Author Contributions

DL and XW conceived and designed the experiments, drafted and interpreted of the present study, and wrote the manuscript. DL and JJ performed the experiments and analyzed the data. All authors critically evaluated and revised the final manuscript.

## Conflict of Interest

The authors declare that the research was conducted in the absence of any commercial or financial relationships that could be construed as a potential conflict of interest.
